# Synthesizing Various Organic Polyacid Compounds for Modifying Forward Osmosis Membranes to Enhance Separation Performance

**DOI:** 10.3390/membranes11080597

**Published:** 2021-08-06

**Authors:** Yi-Li Lin, Bharath Samannan, Kuo-Lun Tung, Jeyabalan Thavasikani, Cheng-Di Dong, Chiu-Wen Chen, Chung-Hsin Wu, Yu-Rong Cheng

**Affiliations:** 1 Department of Safety, Health and Environmental Engineering, National Kaohsiung University of Science and Technology, Kaohsiung 82445, Taiwan; bharathvsb5@gmail.com; 2 Department of Chemistry, Sacred Heart College, Tirupattur 635 601, India; jayabalandr@gmail.com; 3 Department of Chemical Engineering, National Taiwan University, Taipei 106, Taiwan; kltung@ntu.edu.tw; 4 Department of Marine Environmental Engineering, National Kaohsiung University of Science and Technology, Kaohsiung 81157, Taiwan; cddong@nkust.edu.tw (C.-D.D.); cwchen@nkust.edu.tw (C.-W.C.); 5 Department of Chemical and Materials Engineering, National Kaohsiung University of Science and Technology, Kaohsiung 80778, Taiwan; wuch@nkust.edu.tw; 6 Department of Fisheries Production and Management, National Kaohsiung University of Science and Technology, Kaohsiung 811213, Taiwan; yrcheng@nkust.edu.tw

**Keywords:** forward osmosis (FO), hybrid material, organic compound, polyacid, polyamide modification, thin-film nanocomposite (TFN) membrane

## Abstract

In order to overcome the challenges of low permeate flux (J_p_) and the accompanying reverse solute flux (J_S_) during the forward osmosis (FO) membrane separation process, we synthesized four hybrid materials of polyacid-based organic compounds and incorporated them into the selective polyamide (PA) layer to make novel thin-film nanocomposite (TFN) FO membranes. The J_p_ and J_S_ of each membrane were evaluated and used along with membrane selectivity (J_p_/J_S_) as indicators of membrane separation performance. The fabricated and modified membranes were also characterized for ridge and valley surface morphologies with increasing hydrophilicity and finger-shaped parallel channels in the PSf substrate. Moreover, two highly hydrophilic nanoparticles of graphene oxide (GO) and titanium oxide (TiO_2_) were introduced with the hybrid materials for PA modification, which can further enhance the J_p_ of the TFN membranes. The highest J_p_ of the TFN membranes achieved 12.1 L/m^2^-h using 0.1% curcumin-acetoguanamine @ cerium polyacid (CATCP) and 0.0175% GO. The characteristic peaks of the hybrid materials were detected on the membrane surface using attenuated total reflectance Fourier transform infrared (ATR-FTIR) spectroscopy, evidencing successful incorporation of the hybrid materials during membrane modification. Here, we present the novel TFN membranes using hybrid materials for separation applications. The reactions for synthesizing the hybrid materials and for incorporating them with PA layer are proposed.

## 1. Introduction

Forward osmosis (FO) has been a promising technique in the last few decades due to the water scarcity caused by the rapid growth of the global population and environmental changes [1]. FO plays a prominent role in the field of freshwater supply and salt rejection thanks to its extremely low energy requirements and high recovery water quality, for which it uses thin-film composite (TFC)-FO membranes [2]. However, the major challenges of FO technology are low permeate flux and the accompanying reverse solute flux during the osmosis process s because FO applies a high salinity draw solution to provide osmotic pressure as a driving force for the separation process. These challenges need to be overcome before wide application of this technology [3]. In recent years, much effort has been devoted to developing new types of membranes by incorporating emerging nanomaterials, such as silica [4], graphene oxide (GO) [5], hybrid materials [6,7], and metal–organic frameworks (MOFs) [8] to make thin-film nanocomposite (TFN) membranes, which have shown great promise for applications in wastewater treatment [9,10], water recovery [11], and energy production [12]. The common principle of incorporating nanoparticles to modify FO membranes is to achieve high hydrophilicity of the top selective layer, which is beneficial for increasing water permeability.

Polyoxometalate, commonly called polyacid, exists in the form of nanosized inorganic clusters and has much potential for the water treatment process, because of its shifting redox properties and high stability in acidic conditions [13]. The application of transition metals, such as cerium (Ce)-based composites, has been shown to be promising for wastewater treatment and the enhancement of membrane validity [14]. Polyacid is hydrophilic in nature and can be used as a nanofiller with high surface area and low synthetic cost [15]. Ce^3+^ and Ce^4+^ transformations can form superoxide anions and hydroxyl radicals [8,16], and Ce^n+^-based polyacid tends to have increased compound stability and solubility in acidic conditions [8,17]. The chemical properties and mechanical strength of the hybrid materials can be controlled by varying the synthesizing factors, such as the ratio of organic and inorganic compounds and reaction time during the hybridization process, to evenly disperse the nanosized inorganic particles in an organic species [18,19].

Curcumin diketimine (CDT) is a hybrid organic compound with antioxidant properties at neutral and acidic conditions [17,20]. It is also used in membrane bioreactors for antibacterial purposes in wastewater treatment [20,21]. The surface modification of TFC membranes using hybrid materials such as CDT may exhibit better performance without further surface modification of the hybrid materials [22,23]. To the authors’ best knowledge, there is no literature adopting this kind of hybrid material for modifying TFN membranes. Therefore, in this study, we synthesized and modified the selective layer polyamide (PA) of a TFC-FO membrane using the prepared materials based on cerium polyacid (CP), including (1) CP–benzoguanamine (CPB), (2) CP-acetoguanamine (CPA), (3) curcumin-benzoguanamine-CP (CBTCP), and (4) curcumin-acetoguanamine-CP (CATCP) as nanofillers to make TFN membranes. Acetoguanamine refers to 2,4-diamino-6-methyl-1,3,5-triazine and benzoguanamine refers to 2,4-diamino-6-phenyl-1,3,5-triazine. Factors including the dosage of organic-inorganic hybrid materials and dose of additional nanoparticles (GO and TiO_2_) were evaluated for enhancing the performance of TFN membranes.

## 2. Materials and Methods

### 2.1. Chemicals and Reagents

All chemicals were purchased in analytical grade and used as received. Polysulfone (PSf) beads (UDEL P-3500 LCD MB7, Solvay advanced polymers, L.L.C) and N-methyl-2-pyrrolidone (NMP, Macron, USA) were used to prepare the support layer. Polyvinyl pyrrolidone (PVP, Acros, Pittsburgh, PA, USA) and lithium chloride (LiCl, anhydrous > 99%) were employed as additives in the PSf casting solution. Sodium dodecyl sulfate (SDS, Showa, Japan), 1,3,5-benzenetricarbonyl trichloride (TMC, 98%, Tokyo Chemicals Industry Co., Ltd., Tokyo, Japan), and m-Phenylenediamine (MPD, >99%, Acros, New York, NY, USA) were used to prepare the PA-selective layer. Sodium chloride (NaCl) was purchased from Taiwan Biotech. HNO_3_, disodium hydrogen phosphate, ammonium molybdate, ammonium ceric sulphate, benzoguanamine, and acetoguanamine were all purchased from Alfa Aesar (Ward Hill, MA, USA). The nanoparticles of graphene oxide (GO, diameter: 90 nm) and titanium dioxide (TiO_2_, Degussa P-25, diameter: 20 nm) were purchased from UniRegion Bio-Tech (Taiwan) and Showa (Tokyo, Japan) for modifying the active layer of the FO membranes.

### 2.2. Preparation of Hybrid Materials

CP was prepared by mixing ammonium ceric sulphate (molecular weight (MW) 332.24 Da) (1 g, 1 mmol) in 10 mL of DI and ammonium molybdate (MW 1235.86 Da) (2 g, 2 mmol) in 10 mL of DI, and stirring at 50 °C for 10 min [17]. A few drops of 4 N HNO_3_ was added to maintain the pH at 4–5. Then, the solution was vigorously stirred at 400–500 rpm for another 1 h. The precipitates were filtered and washed using DI water, and dried at 50–60 °C in an oven overnight.

Cerium polyacid–benzoguanamine (CPB) was prepared by using the same steps for preparing CP except the solution of benzoguanamine (MW 125.02 Da) (0.125 g, 1 mmol) in 15 mL of ethanol was added dropwise in 1 h of vigorously stirring at 400–500 rpm. The product of a yellowish color was filtered and dried at 50 °C in an oven overnight. Cerium polyacid–acetoguanamine (CPA) was prepared by using the same procedure of preparing CPB, but instead used acetoguanamine (MW 125.13 Da) (0.125 g, 1 mmol) in 15 mL of ethanol.

For preparing curcumin–benzoguanamine cerium polyacid (CBTCP), a mixture of curcumin (MW 368.38 Da) (0.368 g, 1 mmol) and benzoguanamine (MW 187.206) (0.50 g, 2 mmol) with 15 mL of ethanol solution was stirred at room temperature in the presence of two drops of piperidine for 6 h. The solution was concentrated through rotavapour, and the resulting residue was refrigerated overnight. The reddish-yellow products are curcumin–benzoguanamine (CBT), which were filtered and washed with DI water [24]. Then, CBT (0.5 g, 1 mmol) in 15 mL of ethanol was added dropwise for the next 1 h with vigorous stirring at 400–500 rpm. The product was filtered using DI water and dried at 80 °C in an oven overnight to get CBPCP. Curcumin–acetoguanamine cerium polyacid (CATCP) was prepared using a similar procedure for preparing CPA, except that benzoguanamine was replaced with acetoguanamine (MW 125.13) (0.250 g, 2 mmol) in 15 mL of ethanol. The formed precipitate was collected and dried at 80 °C in an oven overnight.

**Figure 1:**
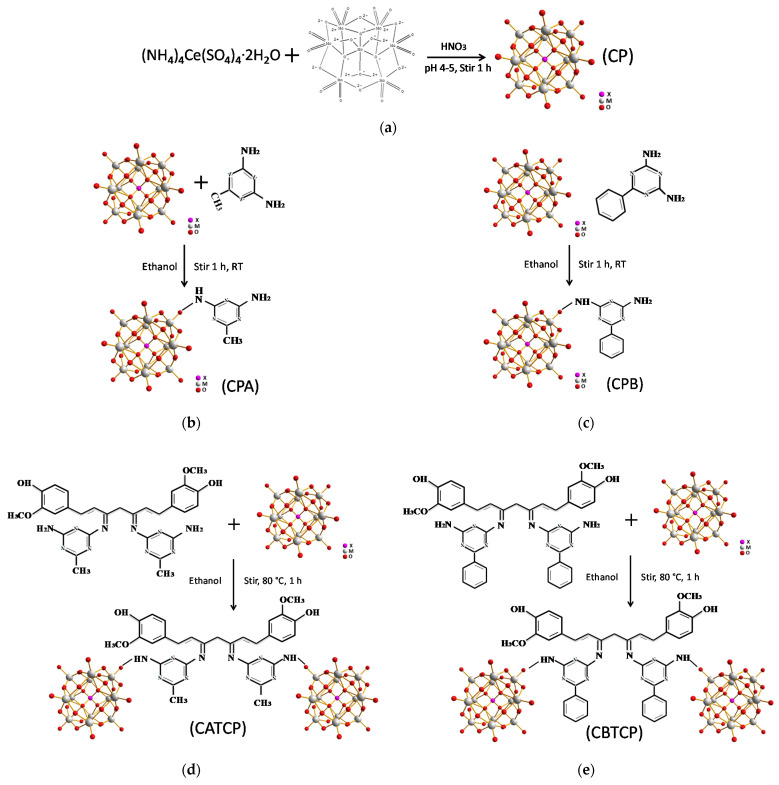
The schematic synthetic procedures and structures of the hybrid materials. (**a**) CP, (**b**) CPA, (**c**) CPB, (**d**) CATCP, and (**e**) CBTCP.

The schematic synthetic procedures and structures of the hybrid materials are presented in Figure 1.

### 2.3. Preparation of FO Membranes

The factors for preparing the virgin TFC membrane were explored in detail in our previous study [25].

#### 2.3.1. Preparation of the PSf Substrate

A polymer solution containing PSf beads (15.5 wt.%), LiCl (3.0 wt.%) and PVP (0.5 wt.%) was dissolved in NMP (81.0 wt.%), stirred for 24 h at 70 °C until it became a smooth and transparent solution, and then degassed for 24 h to remove gases from the liquid and to prevent bubble formation during the process of casting the PSf membrane.

To fabricate the PSf membrane, the solution was poured onto a glass plate at a casting speed of approximately 15 cm/s using a ZUA 2000 Zehntner Universal Film Applicator (Zehntner GmbH Testing Instruments) with the casting height of 300 µm. The glass plate was then immediately immersed into a deionized (DI) water bath (23 °C). The nonsolvent induced phase inversion (PI) method was adopted to form the PSf membranes. The membranes were cleaned using DI water, to remove the excess solvent and additives, and then stored in DI water in the refrigeration (4 °C) for further formation of the PA active layer.

#### 2.3.2. Preparation of the PA Selective Layer

The PA layers, with differing incorporation of the hybrid materials, were formed on the PSf substrates using the interfacial polymerization (IP) approach. The CPA, CPB, CBTCP, and CATCP nanoparticles were separately dosed at different weight ratios (0.05, 0.1, and 0.2 wt.%), in a solution containing 2 wt.% MPD monomer and sodium dodecyl sulphate (SDS) (0.5 wt.%) in DI water, and the solutions were sonicated for 1 h for an even distribution. The PSf substrate was dried at first, then immersed in the MPD solution for 2 min, and taken out to remove excess MPD solution on the membrane surface using a rubber knife. Then, the MPD-saturated substrate was immediately immersed in the TMC solution (0.15 wt.% in n-hexane) for 3 min. The experimental conditions of the PA layer, to prepare the TFN membranes with different hybrid materials, are listed in Table 1. To further modify the PA layer, high hydrophilic GO (0.0175 wt.%) and TiO_2_ (0.1 wt.%) nanoparticles were dosed with hybrid materials in the experimental conditions summarized in Table 2.

### 2.4. FO Filtration Experiments

The virgin (TFC) and modified (TFN) membranes were installed in a self-designed crossflow FO filtration model [26] to evaluate the performance of FO membranes in terms of permeate flux (J_p_; L/m^2^-h, LMH) and reverse salt flux (J_s_; mole/m^2^-h, nMH) at room temperature (25 °C). DI water and 1 M NaCl solution were used as the feed solution (FS) and draw solution (DS), respectively. J_P_ and J_S_ were calculated using the following Equations (1) and (2), respectively. The reverse salt flux was measured using the volumetric and concentration changes of the solution depending on the conductivity measurement [22].

(1) $$J_{p}=\frac{\Delta V}{A_{m}\Delta t}$$

(2) $$J_{s}=\frac{C_{f,t}\times J_{f,t}-C_{f,o}\times V_{f,o}}{A_{m}\times \Delta t}$$

where ΔV (L) is the volume of the permeated DI water from FS to DS during the experimental time period Δt (h); A_m_ is the effective surface area of the membrane; C_f,t_ and J_f_,_t_ are the concentration and permeate flux of FS at time t, respectively; C_f,o_ and V_f,o_ are the initial concentration and volume of FS, respectively.

### 2.5. Analytical Methods

All the samples were completely dried before conducting the following analyses. The functional groups of the hybrid materials were analyzed using a Fourier transform infrared spectrometer (FTIR, Spectrum 100, PerkinElmer, Waltham, MA, USA), and those of the TFC and TFN membranes were analyzed using an attenuated total reflectance FTIR (ATR-FTIR) (Spectrum 100, PerkinElmer). FTIR and ATR-FTIR analyses were both performed at room temperature over the wave number range of 450–3500 cm^−1^ with a resolution of 4 cm^−1^, and the spectrum of the averaged results of 40 scans of each sample was reported. Contact angles were measured to represent membrane surface hydrophilicity, according to the standard sessile drop method, by using a contact angle meter (Phx mini, Phoenix, Korea). The contact angles of each membrane sample were reported as the average of at least five droplets of Milli-Q water applied at random sites. The morphology and chemical compositions of the synthesized hybrid materials and membrane surfaces were investigated using a scanning electron microscope (SEM; SU-5000, Hitachi, Japan) and energy dispersive X-ray spectroscopy (EDS), respectively, after sputtering a thin layer of Au on the sample surface to enhance conductivity. The particle size of each hybrid material was measured using a laser particle size analyzer (90 Plus, Brookhaven Instruments Co., New York, NY, USA).

## 3. Results and Discussion

### 3.1. Characterization of the Hybrid Materials

**Figure 2:**
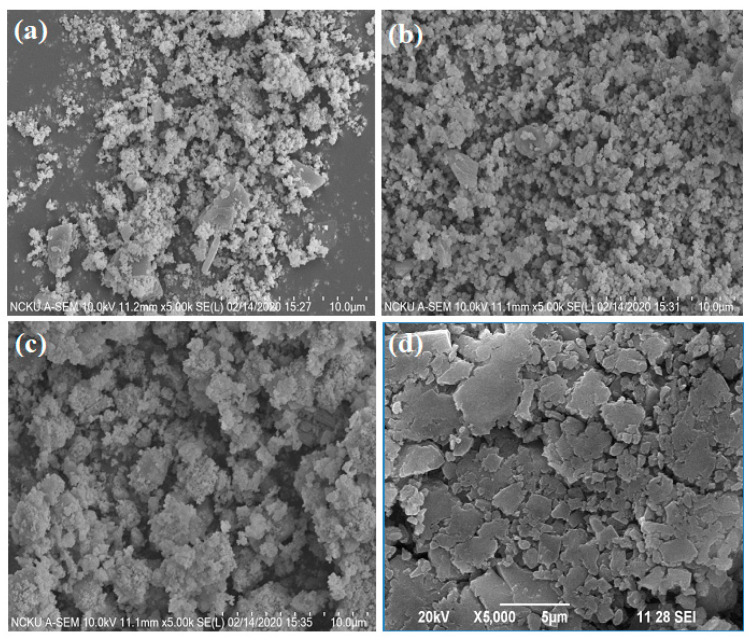
SEM images of the hybrid materials of (**a**) CPA, (**b**) CPB, (**c**) CATCP, and (**d**) CBTCP.

The morphology and chemical compositions of the synthesized hybrid materials are displayed in Figure 2 and Table 3, respectively. SEM images revealed that the different hybrid materials were composed of agglomerate nanoparticles with approximate diameters of 105–155 nm, measured using Image J software (Version 1.53k), and elemental mapping shows the presence of elements of the organic and inorganic compounds of the hybrid materials. The intensive peaks of the carbon (C), nitrogen (N), and oxygen (O) elements indicate the possible presence of CDT. The analyzed EDS spectra indicate the presence of molybdenum (Mo) and O that constitute CP, as summarized in Table 3. Moreover, the elemental composition of CPA was analyzed as O: 29.89%, N: 0.38%, Mo: 63.84%, and C: 5.89% (Table 3), which corresponds well to the theoretical gravimetric value of Mo (57.02%) [17]. The elemental composition of CPB was analyzed as O: 22.31%, N: 2.12%, Mo: 54.37%, and C: 21.18% (Table 3), which corresponds well to the theoretical gravimetric value of Mo (55.59%) [17,27]. The elemental composition of CATCP was analyzed using EDS as O: 27.47%, N: 0.24%, Mo: 61.85%, and C: 10.43% (Table 3), which corresponds well to the theoretical gravimetric value of Mo (60.63%) [17,28]. The elemental composition of CBPCP was analyzed using EDS as O: 22.77%, N: 2.35%, Mo: 55.55%, and C: 19.33% (Table 3), which corresponds well to the theoretical gravimetric value of Mo (54.85%) [17,23]. The above analytical results validate the successful synthesis of the hybrid materials.

**Figure 3:**
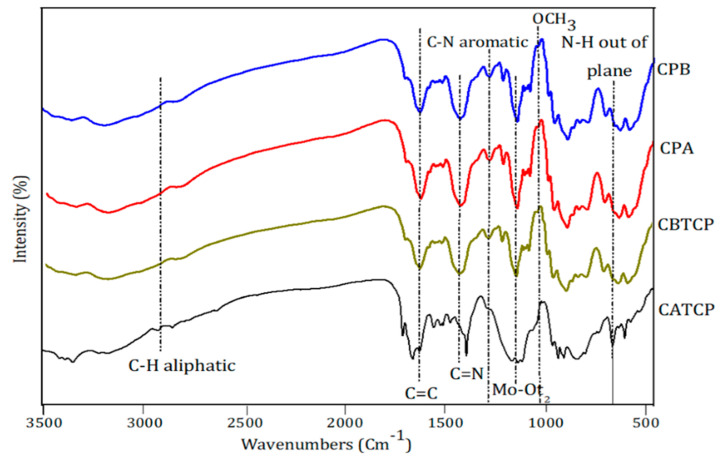
FTIR spectral of the hybrid materials of CPA, CPB, CBTCP and CATCP.

The FTIR spectra of CPA, CPB, CATCP, and CBTCP are presented in Figure 3. The band at 1138 → 1192 cm^−1^ indicates the presence of Mo–O_t1_, and the one at 1100 → 1110 cm^−1^ represents the stretching vibration of Mo–O_t2_.The stretching frequency at 1063 → 1076 cm^−1^ is observed in the band of Mo–O_b_ (intra), and a Mo–O_b_ (inter) band is observed at 954 → 960 cm^−1^. Those at 798 → 822 cm^−1^ and 547 → 590 cm^−1^ indicate the bands of M–O–M and M–N (N = transition metal) [17]. The tentative assignments of the hybrid materials are as follows; the bands of 3146, 1653, 1542, and 1409 cm^−1^ indicate a ν (C–H) aromatic ring, ν (N–H) 1° vibration, ν (N–H) 3° vibration, and C–C aromatic ring, respectively. The band at 641 → 697 cm^−1^ indicates the ν (N–H) out of plane [24]. The stretching vibration of ν (C–H) causes the band at 2914 cm^−1^. The frequencies at 1505, 1273, and 1014 cm^−1^ display ν (C=O, C=C), ν (C=O) phenolic, and ν (OCH_3_) groups. A band of CPB at 1280 cm^−1^ indicates the stretching vibration of ν (C–N) aromatic rings [23,29]. The positive shift in the vibrations of polyacid over the organic compound benzoguanamine may be due the structural modification of adding triazine ring on the guest molecule. CPB has bands at 1542, 1653 and 690 cm^−1^ that are attributed towards three amine functional groups, and N-H was constructed via N^3+^−H^3^⋯O^2–^ with charge interaction. The bands at 822 and 1414 cm^−1^ are attributed to (C-N) and (C=N) of characteristic groups of s-triazine (benzoguanamine) in CBTCP [30]. All hybrid material exhibits the presence of bands around 3400 cm^−1^, which are O–H vibrations. The peak at 2927 cm^−1^ is corresponding to the aliphatic C-H stretching band of the acetoguanamine in the CPA and CATCP hybrid materials [27].

### 3.2. Performance of the TFC and TFN Membranes

**Figure 4:**
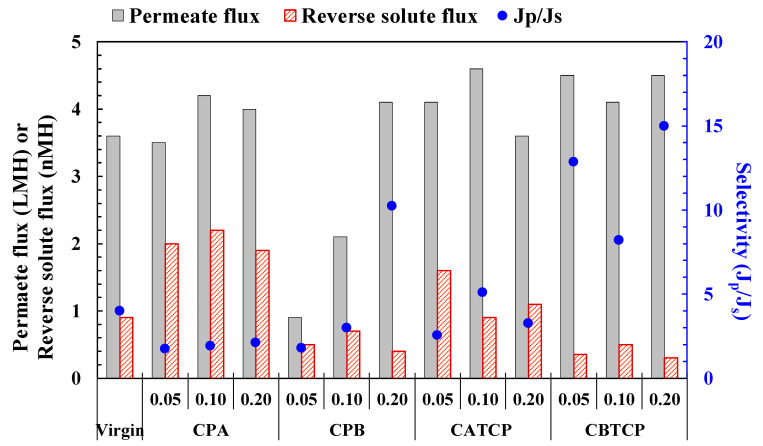
FO performance of the TFC and TFN membranes using hybrid materials with different dosages (%). The experimental conditions are presented in Table 1.

The performance of the virgin modified FO membrane are presented in Figure 4. An enhancement in water flux occurred upon the addition of CPA (0.05–0.20 wt.%), CPB (0.2 wt.%), CATCP (0.05–0.10 wt.%), and CBTCP (0.05–0.20 wt.%), which can be explained by the increased surface hydrophilicity on the TFN membranes [10,13,20] with results presented and discussed in the following Section 3.4.3. Moradi et al. prepared a polyethersulfone membrane by incorporating curcumin functionalized boethmite (B-Cur) nanoparticles, which also exhibited enhanced water flux, metal rejection, and antifouling properties [20]. The relatively higher permeate flux for the TFN membranes modified using CATCP and CBTCP, as compared to those using CPA and CPB, may be due to the presence of hydrophilic hydroxyl groups in the structures of CATCP and CBTCP (Figure 1). However, for several TFN membranes with PA modified using CPA and CPB, the increase in J_p_ is accompanied by an increase in J_s_, resulting in a decrease in membrane selectivity (J_p_/J_s_). This phenomenon may be due to improper or loose binding of the hybrid materials with the PA polymer that became a defect on the membrane surface for water and salt penetration. Among the evaluated membranes, the incorporation of 0.10 wt.% CATCP in PA formation resulted in the membrane with the highest permeate flux (4.6 LMH) and moderate selectivity (5.1), and the incorporation of 0.20 wt.% CBTCP in PA formation resulted in the membrane with the second highest permeate flux (4.5 LMH) and the highest selectivity (15.0) [20]. Compared to the performance of the other TFN membranes—prepared using exactly the same materials of PA and PSf but different nonfilters, namely, fumed silica (SiO_2_), dried SiO_2_, and 3-aminopropyltriethoxysilane (APTES)-modified SiO_2_—the TFN membranes modified using CPA and CPB exhibited similar separation performance to those modified using SiO_2_ nanofillers, and the TFN membranes modified using CATCP and CBTCP exhibited superior separation performance [31].

On the other hand, Ghorbani et al. [13] reported significantly enhanced FO performance by embedding supramolecular star polymers into the GO-active layer of an FO membrane. After considering that the incorporation of additional nanoparticles in the cross-linking of the selective layer may further enhance separation performance [13], the two better-performing hybrid materials (CATCP and CBTCP) were selected to be studied with further incorporation of other hydrophilic compounds, namely GO and TiO_2_, which aimed to enhance the performance of the TFN membranes. The results will be discussed in the following Section 3.3.

### 3.3. Performance of the TFN Membranes with Additional Incorporation of GO and TiO_2_

**Figure 5:**
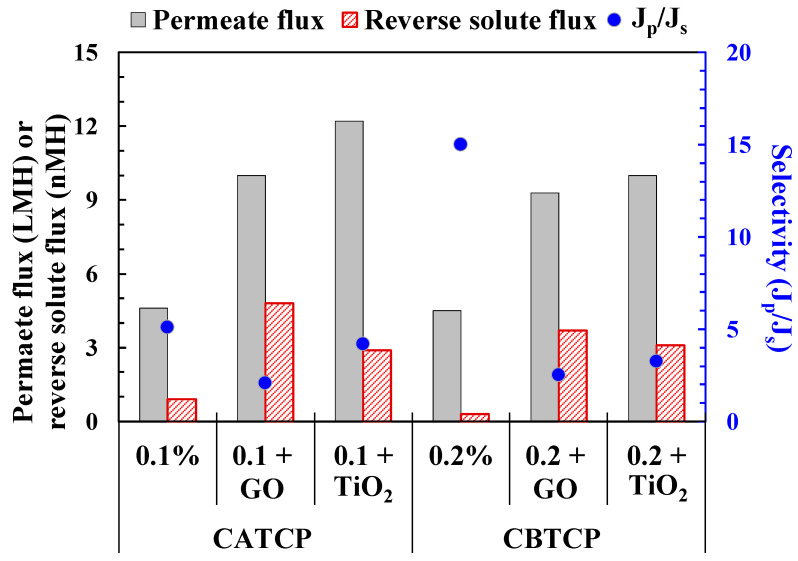
FO performance of TFN-FO membranes using CPA, CPB, CATCP, and CBTCP with additional dosage of GO, and TiO_2_. The experimental conditions are presented in Table 2.

Figure 5 presents the performance of the TFN membranes (with the better-performing hybrid materials, CATCP and CBTCP) with additional incorporation of 0.0175 wt.% GO and 0.10 wt.% TiO_2_. The addition of highly hydrophilic GO and TiO_2_ may enhance the surface hydrophilicity of the TFN membranes so as to increase permeate flux; the results in Figure 5 revealed a significant increase and validated this speculation. However, the addition of GO and TiO_2_ also caused a moderate increase in the reverse solute flux, resulting in a slight-to-considerable decrease in membrane selectivity (blue dots in Figure 5). This phenomenon may be explained by the fact that the dose of GO and TiO_2_ along with CATCP and CBTCP nanoparticles may interfere with the interfacial polymerization of PA, leading to the formation of defects on the membrane surface which cause easier penetration of salt and water. This has been reported for PA modification of other TFC membranes [32,33]. Moreover, the aggregation of nanoparticles may occur on the membrane surface, which has been suggested to have considerable effects on membrane performance [34] and will be discussed in Section 3.4.1. It is worth noting that the dosage of GO is less than one-fifth of that of TiO_2_, but also exhibits a remarkable increase in permeate flux. The reason is that dosing GO into membranes can create additional capillaries which allow the quick passage of water molecules [35]. After considering that the TFN membranes incorporating CATCP and CBTCP along with TiO_2_ both exhibited higher permeate flux and selectivity than those along with GO, the membranes with TiO_2_ addition were selected for surface characterization using ATR-FTIR, SEM, EDS, and contact angle measurements. The results will be presented in the following Section 3.4.

### 3.4. Characterization of the TFN Membranes

#### 3.4.1. Surface Morphology and Composition

**Figure 6:**
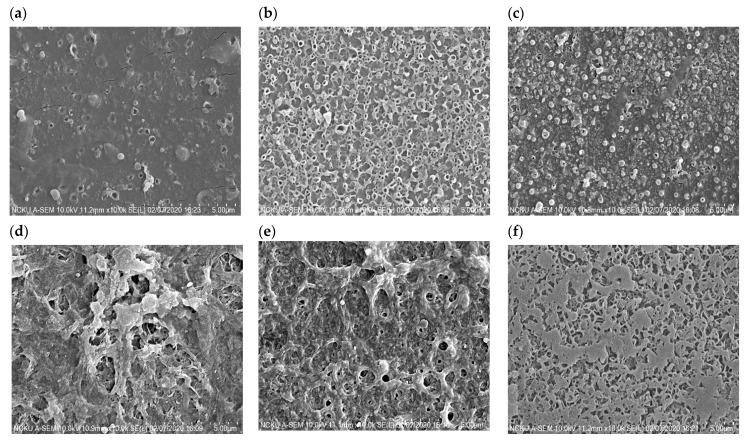
Top surface morphologies of the TFN membranes using (**a**) CPA (0.2 wt.%), (**b**) CPB (0.2 wt.%), (**c**) CATCP (0.1 wt.%), (**d**) CBTCP (0.2 wt.%), (**e**) CATCP—TiO_2_ (0.1 and 0.1 wt.%, respectively), and (**f**) CBTCP—TiO_2_ (0.2 and 0.1 wt.%, respectively).

The surface morphology of the TFN membranes is presented in Figure 6. SEM images in Figure 6 reveal that the top surface of the TFN membranes has the peak-and-valley characteristic structures of the PA layer, created through the IP reaction of TMC and MPD monomers at the organic–inorganic solvent interface [36]. However, some aggregation of nanoparticles can be observed in the valley area of TFN membranes, leading to a smooth membrane surface with decreasing surface roughness. Although the structures of CPA and CPB are similar (Figure 1a,b), their dispersion on the PA layer varied obviously. CPA nanoparticles aggregated considerably on the membrane surface (Figure 6a) while CPB nanoparticles dispersed evenly (Figure 6b), which explains the higher permeate flux and selectivity of the TFN membrane as modified using CPB, rather than using CPA, as shown in Figure 4. Similar correlations between the surface morphology and membrane performance of the TFN membranes using CATCP and CBTCP nanoparticles with similar structures (Figure 1d,e) are also observed. Although the dosage of CATCP is a half of that of CBTCP for preparing TFN membranes (0.1 vs. 0.2 wt.%), significant particle aggregation of CATCP was observed on the membrane surface (Figure 6c), which may explain its lower selectivity than the TFN membrane using CBTCP (Figure 4). As for the TFN membrane with further addition of TiO_2_, considerably less particle aggregation was observed on the surface of the CATCP-TiO_2_ membrane (Figure 6e) as compared to that of the CBTCP-TiO_2_ membrane (Figure 6f), which corresponded to the higher permeate flux and selectivity of the TFN membrane using CATCP-TiO_2_ (Figure 4). Overall, it can be concluded that particle aggregation on the PA layer has a significant effect on membrane performance, regardless of the physico-chemical characteristics of the dosed nanoparticles. Therefore, a proper preparation procedure, and adjustments to increase the even dispersion of nanoparticles, are worthy of further exploration in order to continue to enhance membrane separation performance.

**Figure 7:**
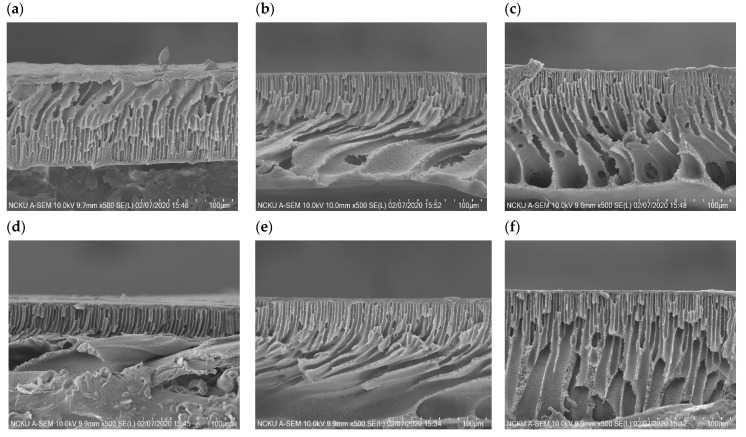
SEM morphologies of the cross sections of the TFN membranes using (**a**) CPA (0.2 wt.%), (**b**) CPB (0.2 wt.%), (**c**) CATCP (0.1 wt.%), (**d**) CBTCP (0.2 wt.%), (**e**) CATCP—TiO_2_ (0.1 and 0.1 wt.%, respectively), and (**f**) CBTCP—TiO_2_ (0.2 and 0.1 wt.%, respectively).

It has been reported that the structure and thickness of the PSf substrate can considerably affect the performance of FO membranes [37]. Therefore, the cross-section of the TFN membranes were observed using SEM, and the results are displayed in Figure 7. It can be seen that each membrane substrate has a similar thickness (75–90 μm), with highly porous and well-developed finger-shaped structures, which can provide fluent channels which do not hinder water molecules, allowing them to achieve a high permeate flux and also to avoid severe internal concentration polarization in the substrate [15,25].

The TFN membranes were also analyzed using ESD to evaluate the composition of characteristic elements of the hybrid materials and TiO_2_ nanoparticles, including C, N, O, Mo, and Ti, and the results are summarized in Table 4. The detection of the characteristic elements confirmed successful incorporation of the hybrid materials and TiO_2_ into the TFN membranes.

#### 3.4.2. Surface Functional Groups

**Figure 8:**
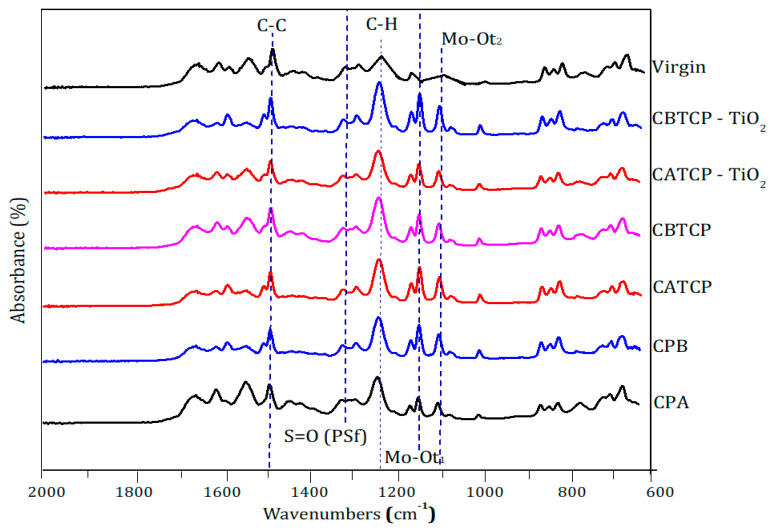
ATR-FTIR of the TFC and TFN membranes.

The ATR-FTIR spectra of TFC and TFN membranes are depicted in Figure 8. The tentative assignments of CP in different organic compounds can be referred to those reported in a previous literature [38]. The band at 1151 → 1171 cm^−1^ indicates the presence of Mo–O_t1_, and that at 1104 → 1107 cm^−1^ shows the stretching vibration of Mo–O_t2_. The tentative assignments of the hybrid materials are as follows: CDT bands of 1651 → 1654 and 1537 → 1544 cm^−1^ indicate the ν (C–H) aromatic ring and ν (N–H) vibration, respectively. The bands at 1249 → 1254 cm^−1^ indicates the stretching vibrations of ν (C–H) aromatic rings. The frequencies at 1491 and 1014 → 1017 cm^−1^ show the ν (C-C) and ν C=O (OCH_3_), and that at 1317 → 1327 cm^−1^ indicate the stretching vibration of ν (S=O) of the PSf substrate. Accordingly, successful incorporation of the hybrid materials in the PA layer can be confirmed.

#### 3.4.3. Surface Hydrophilicity

**Figure 9:**
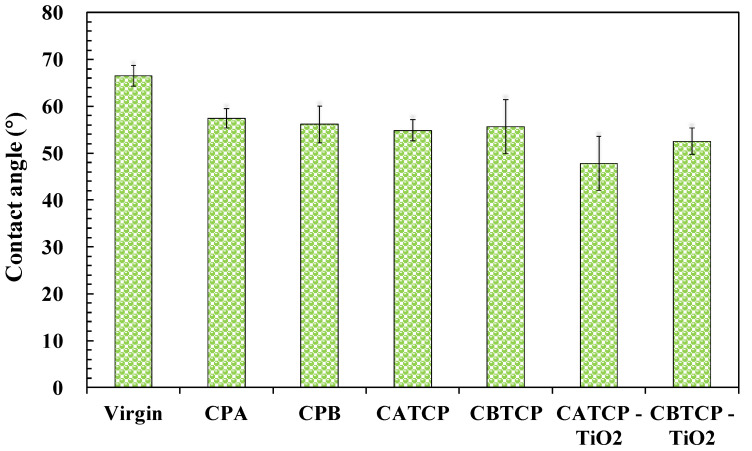
Contact angle measurements of the TFC and TFN membranes. The experimental conditions of TFN membranes are the same as those in Figure 6 and Figure 7.

The hydrophilicity of the membrane surface can be explained using the results of contact angle measurements, and the results are displayed in Figure 9. It is obvious that the hydrophilicity of the TFN membranes significantly increased as compared to the virgin TFC membrane, especially those with an additional dose of highly hydrophilic TiO_2_ nanoparticles, which correlated well with increasing permeate flux of the TFN membranes in Figure 4 and Figure 5. Similar phenomena were reported for membrane modification using hydrophilic materials such as polyoxometalates [11], GO [25], TiO_2_ [12,39], and B-Cur [20]. Therefore, the incorporation of the four synthesized hybrid materials in this study, along with TiO_2_, can increase the wettability of FO membranes [11], maintain low reverse solute flux, and conserve satisfactory membrane selectivity.

**Figure 10:**
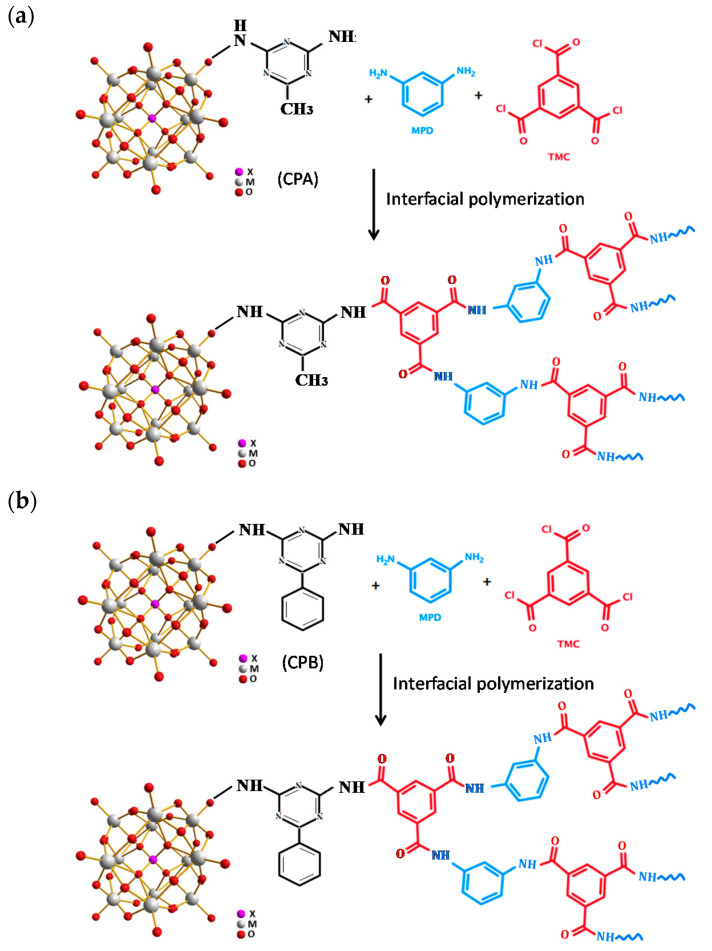

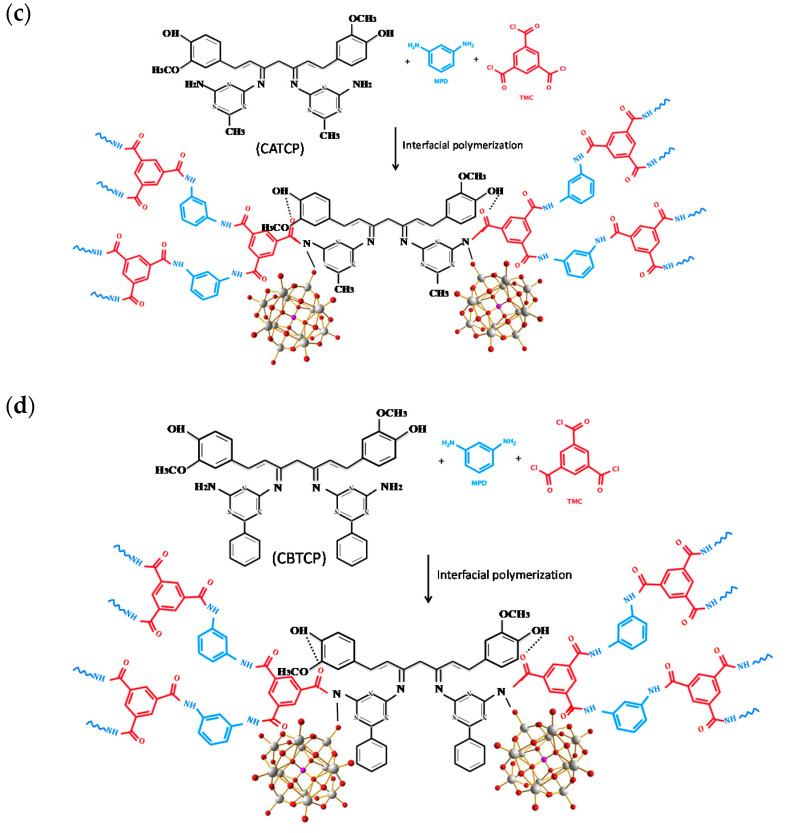
The proposed reactions between CPA, CPB, CATCP, and CBTCP during the interfacial polymerization of PA layer.

The possible reactions between CPA, CPB, CATCP, and CBTCP during the interfacial polymerization of PA layer are proposed in Figure 10. Considering the sub-microscale of the hybrid materials without an abundant amount of hydrophilic functional groups (such as hydroxyl groups) in their structure (Figure 1 and Figure 2), it is speculated that water molecules can penetrate through the spaces between the synthesized polymer chains and those in the structure of hybrid materials, which has been previously reported for membranes modified using metal–organic frameworks [40,41].

## 4. Conclusions

In this study, four hybrid materials were synthesized and used as novel nanofillers for modifying the surface PA layer to make TFN-FO membranes. Successful incorporation of the hybrid materials on the membrane surface was characterized using SEM, EDS, ATR-FTIR, and contact angle measurements, and the possible reactions between CPA, CPB, CATCP, and CBTCP during the interfacial polymerization of PA layer were proposed. It is speculated that water molecules can penetrate through the spaces between the synthesized polymer chains and those in the structure of the hybrid materials, resulting in an increased permeate flux and surface hydrophilicity of the TFN membranes. The additional incorporation of hydrophilic GO and TiO_2_ nanoparticles further increased the permeate flux of TFN membranes, while maintaining low reverse salt flux and satisfactory membrane selectivity. The highest J_p_ of the TFN membranes achieved 12.1 LHM using 0.1% CATCP and 0.0175% GO. Thus, we present these new TFN membranes, which use hybrid materials, for separation applications.

## 5. Patents

This paper is without patents, as reported in the manuscript.

## Figures and Tables

**Table 1:** Experimental conditions of the PA layer of the TFN membranes.

Hybrid Material	Dosage (wt.%)	Aqueous Solution			n-Hexane
		MPD (wt.%)	SDS (wt.%)	DI Water (wt.%)	TMC (*w*/*v*.%)
CPA, CPB, CBTCP CATCP	0	2.0	0.1	97.90	0.15
	0.05	2.0	0.1	98.85	0.15
	0.10	2.0	0.1	97.80	0.15
	0.20	2.0	0.1	97.70	0.15

**Table 2:** Experimental conditions of the PA layer of the TFN membranes with additional dosage of GO or TiO_2_.

Hybrid Material	Nanoparticle (wt.%)	Aqueous Solution			n-Hexane
		MPD (wt.%)	SDS (wt.%)	DI Water (wt.%)	TMC (*w*/*v*.%)
CBTCP	GO: 0.0175	2.0	0.1	97.68	0.15
(0.2 wt.%)	TiO_2_: 0.1	2.0	0.1	97.60	0.15
CATCP	GO: 0.0175	2.0	0.1	97.78	0.15
(0.1 wt.%)	TiO_2_: 0.1	2.0	0.1	97.70	0.15

**Table 3:** EDS analysis of the hybrid materials (powder samples).

Hybrid Material	Carbon (wt.%/at.%)	Nitrogen (wt.%/at.%)	Oxygen (wt.%/at.%)	Molybdenum (wt.%/at.%)
CPA	5.89/16.05	0.38/0.89	29.89/61.24	63.84/21.81
CPB	21.18/45.49	2.12/3.91	22.31/35.97	54.37/14.62
CATCP	10.43/26.74	0.24/0.52	27.47/52.87	61.85/19.85
CBTCP	19.33/36.4	2.35/0.57	22.77/52.80	55.55/19.50

**Table 4:** EDS analysis of TFN membranes.

Hybrid Material	Carbon (wt.%/at.%)	Nitrogen (wt.%/at.%)	Oxygen (wt.%/at.%)	Molybdenum (wt.%/at.%)	Ti (wt.%/at.%)
CPA	51.33/78.18	0.96/1.27	11.83/13.65	35.88/6.90	-
CPB	54.14/77.57	2.73/3.35	12.66/13.62	30.48/5.47	-
CATCP	51.30/74.20	5.22/6.47	12.53/13.60	30.27/5.48	0.68/0.25
CBTCP	52.51/74.34	6.02/7.31	12.40/13.18	28.96/5.13	0.12/0.04

## Data Availability

This study did not report any data in a public dataset, whether analyzed or generated.
